# Protocol for a prospective cohort study to determine the multimodal biomarkers of delirium and new dementia after acute illness in older adults: ORCHARD-PS

**DOI:** 10.1136/bmjopen-2025-102028

**Published:** 2025-06-13

**Authors:** Jasmine Ming Gan, Lily Elderton, Meenu Vijayakumar Sheela, Jessica Knight, John Louca, Sarah Evans, Kinza Shahab, Nicola G Lovett, Mary Sneade, Nycola Muchenje, Mariya Fenchyn, Davide Simonato, Aubretia McColl, Sarah Tamsin Pendlebury

**Affiliations:** 1 Wolfson Centre for Prevention of Stroke and Dementia, Nuffield Department of Clinical Neurosciences, University of Oxford, Oxford, UK; 2 Neurosciences Research Delivery Team (DENDRON), Oxford University Hospitals NHS Foundation Trust, Oxford, UK; 3 Oxford (Thames Valley) Foundation School, Oxford University Hospitals NHS Foundation Trust, Oxford, UK; 4 Departments of Acute General Internal Medicine and Geratology, Oxford University Hospitals NHS Foundation Trust, Oxford, UK; 5 University Department of Elderly Care, Royal Berkshire NHS Foundation Trust, Reading, UK; 6 Department of Neuroradiology, Oxford University Hospitals NHS Foundation Trust, Oxford, UK

**Keywords:** Delirium, Dementia, Clinical Protocols, GERIATRIC MEDICINE, INTERNAL MEDICINE, Cognition

## Abstract

**Introduction:**

Delirium is common in the older hospital population and is often precipitated by acute illness. Delirium is associated with poor outcomes including subsequent cognitive decline and dementia and may therefore be a modifiable risk factor for dementia. However, the mechanisms underpinning the delirium–dementia relationship and the role of coexisting acute illness factors remain uncertain. Current biomarker studies of delirium have limitations including lack of detailed delirium characterisation with few studies on neurodegenerative or neuroimaging biomarkers especially in the acute setting. The Oxford and Reading Cognitive Health After Recovery from acute illness and Delirium—Prospective Study (ORCHARD-PS) aims to elucidate the pathophysiology of delirium and subsequent cognitive decline after acute illness in older adults, through acquisition of multimodal biomarkers for deep phenotyping of delirium and acute illness, and follow-up for incident dementia.

**Methods and analysis:**

ORCHARD-PS is a bi-centre, prospective cohort study. Consecutive eligible patients requiring acute hospital admission or assessment are identified by the relevant acute clinical care team. All patients age >65 years without advanced dementia, nursing home residence, end-stage frailty or terminal illness are eligible. Details of potential participants are communicated to the research team and written informed consent or consultee agreement is obtained. Participants are interviewed as soon as possible after admission/assessment using a structured proforma.

Data are collected on demographics, diagnosis and comorbidities, social and functional background. Delirium is assessed using the 4A’s test, Confusion Assessment Method (long-form), Observational Scale of Level of Arousal, Richmond Agitation-Sedation Scale and Memorial Delirium Assessment Scale and diagnosed using the Diagnostic and Statistical Manual of Mental Disorders, Fifth Edition criteria. Delirium is categorised by time of onset (prevalent vs incident), dementia status, motoric subtype, severity and duration. Cognitive tests include the 10-point Abbreviated Mental Test and Montreal Cognitive Assessment. Participants are reassessed every 48–72 hours if remaining in hospital. Informant questionnaire data and interview are supplemented by hand searching of medical records and linkage to electronic patient records for nursing risk assessments, vital observations, laboratory results and International Classification of Diseases, Tenth Revision diagnostic and procedure codes.

In-person follow-up with more detailed cognitive testing and informant interview is undertaken at 3 months, and 1 and 3 years supplemented with indirect follow-up using medical records. Blood banking is performed at baseline and all follow-ups for future biomarker analyses. CT-brain and MRI-brain imaging acquired as part of standard care is obtained for quantification of brain atrophy and white matter disease/stroke supplemented by research CT-brain imaging. Outcomes include length of hospitalisation, change in care needs, institutionalisation, mortality, readmission, longitudinal changes in cognitive and functional status and incident dementia. Biomarker associations with delirium, and with incident dementia on follow-up, will be determined using logistic or Cox regression as appropriate, unadjusted and adjusted for covariates including demographics, baseline cognition, frailty, comorbidity and apolipoprotein E genotype.

**Ethics and dissemination:**

ORCHARD-PS is approved by the South Central—Berkshire Research Ethics Committee (REC Reference: 23/SC/0199). Results will be disseminated through peer-reviewed publications and conference presentations.

**Trial registration number:**

ISRCTN24171810.

**STRENGTHS AND LIMITATIONS OF THIS STUDY**

- The Oxford and Reading Cognitive Health After Recovery from acute illness and Delirium—Prospective Study includes a deeply phenotyped cohort of older acute hospital patients with detailed prospective delirium ascertainment and multimodal (clinical, blood, digital, neuroimaging) biomarkers.
- Cognitive and delirium assessments are repeated every 48–72 hours by the study team to assess for new delirium and change in health status.
- Study team assessments are not performed every day but daily clinical care team review and review of records minimise missed delirium.
- Participants undergo in-person follow-up at 3 months, 1 and 3 years for evaluation of longitudinal cognitive and functional change.
- Attrition from follow-up will occur and will be more likely in older, multimorbid patients at higher risk of dementia, but this is mitigated by indirect follow-up using medical records.

## Introduction

**Figure 1:**
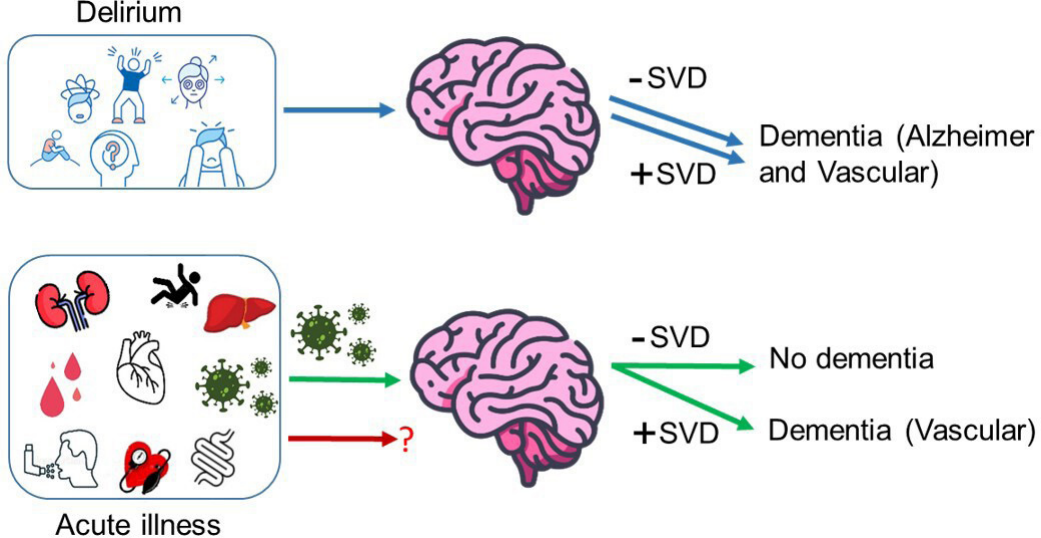
Schematic diagram showing how two specific acute illness features with presumed different systemic responses impact future dementia risk according to the underlying brain pathology: delirium increases dementia risk irrespective of brain imaging findings (blue arrows) whereas infection only increases dementia risk in those with pre-existing small vessel disease (SVD, green arrows). The role of other acute illness features including hypoxia and inflammation without infection is currently unknown (red arrow).

 In the UK, older people aged >65 years occupy over half of hospital emergency bed days.^1^ Cognitive frailty, including delirium, dementia and objective cognitive deficits, is common in older hospitalised patients and affects over one-third with unplanned admission to acute medical services.^2^ Delirium, characterised by an acute and fluctuating change in attention, awareness and cognition, is often precipitated by acute illness. The cognitive impact of delirium extends beyond the immediate period in-hospital, and it is associated with long-term cognitive decline and subsequent dementia.^3^,^5^ Similarly, features of acute illness and emergency hospitalisation, specifically infection, are associated with increased risk of dementia.^5 6^ Delirium and infection are frequently coassociated but recent research indicates that they are independent risk factors for dementia with dose–response effects.^5^ Importantly, however, infection is only a risk factor in those with underlying cerebral small vessel disease, whereas delirium is a risk factor irrespective of the underlying brain imaging findings (figure 1). Furthermore, delirium appears a more important risk factor for dementia in older people, whereas infection seems more important in younger patients.

Delirium and infection may therefore be potentially modifiable risk factors for dementia. Current delirium management strategies are focused on multicomponent interventions for delirium prevention and drugs to manage delirium symptoms.^7^ However, effective treatment to reduce delirium duration, severity and adverse outcomes (including subsequent dementia) remains limited.^8 9^ Infection causes a pronounced systemic response including inflammation but the specific mechanisms underpinning the relationship with dementia are unknown. Furthermore, the impact of other acute illness features such as hypoxia on dementia risk is unclear. Studies to elucidate the pathophysiology underlying delirium, acute illness features and subsequent dementia are therefore required to identify therapeutic targets.

Biomarkers are crucial in advancing our knowledge of the relationship between delirium and dementia and could be used as a tool for risk stratification. A recent systematic review of 113 studies on delirium biomarkers found important limitations.^10^ First, most studies assessed inflammatory biomarkers in the blood and CSF. Only 20% of studies examined markers of neurodegeneration including the β-amyloid, pathologic τ and biomarkers of neurodegeneration or neuronal injury (ATN) and none measured all three ATN biomarkers together. Second, neuroimaging biomarkers were assessed in less than 10% of studies, which all used MRI or positron emission tomography scan. Third, the majority of studies binarised delirium as present or absent and did not characterise delirium subtype (14%), severity (19%) or duration (11%), and the biomarker profile of different delirium characteristics and associations with long-term cognitive decline are unknown. Finally, few studies assessed multimodal biomarkers combining clinical, blood and neuroimaging markers necessary to understand the interplay between the systemic response to acute illness, neuroinflammation and underlying brain changes in delirium and dementia risk.

In this protocol, we therefore describe the methodology for a bi-centre, prospective cohort of older acute hospital patients with acquisition of multimodal biomarkers for deep phenotyping of delirium and acute illness, and follow-up for subsequent dementia.

## Aims and objectives

The primary aim of the Oxford and Reading Cognitive Health After Recovery from acute illness and Delirium—Prospective Study (ORCHARD-PS) is to elucidate the pathophysiology of delirium and subsequent cognitive decline following acute illness in older people.

The main objectives are to:

1. Determine the multimodal (clinical, bloods, neuroimaging, digital) biomarker associations with delirium and new dementia to 3-year follow-up.
2. Determine the predictors of poor outcome in patients with delirium, including length of stay, institutionalisation, readmission to hospital, cognitive decline and mortality.
3. Assess the potential of advanced photon-counting CT brain imaging in providing enhanced information through better grey-white matter differentiation, spatial resolution and quantification of brain atrophy.

## Methods and analysis

### Study design and setting

ORCHARD-PS is an observational cohort study conducted at two sites in the United Kingdom: the Oxford University Hospitals NHS Foundation Trust (OUHFT), Oxford and the neighbouring Royal Berkshire NHS Foundation Trust (RBFT), Reading. The OUHFT is a large teaching hospital trust comprising four hospitals (John Radcliffe Hospital, Nuffield Orthopaedic Centre, Churchill Hospital in Oxford and the Horton General Hospital in Banbury) and provides acute hospital care to a population of >8 00 000 in Oxfordshire. The RBFT includes one large acute hospital, the Royal Berkshire Hospital serving a population of >5 00 000 people in the Berkshire region. Both hospital trusts offer acute general medicine services and acute ambulatory care services (same-day emergency care-SDEC). The combined Oxfordshire and Berkshire populations are representative of the background population in England with an urban/rural mix and although overall less deprived than the average in England, all levels of deprivation are represented. ORCHARD-PS is approved by the South Central—Berkshire Research Ethics Committee (REC Reference: 23/SC/0199). The recruitment for ORCHARD-PS began on 9 August 2023 and is scheduled to finish on 31 July 2025, with in-person study follow-up ending on 28 February 2028.

### Participants and eligibility

Consecutive eligible patients are identified from the acute clinical care teams including the acute general medicine and SDEC teams. Participants are recruited as soon as possible after admission or SDEC assessment.

#### Inclusion

Patients aged >65 years requiring acute hospital admission or assessment in SDEC are eligible. Written or verbal informed consent is required from the patient, or consultee agreement (relative, friend or healthcare professional) for patients lacking capacity to give informed consent. Verbal consent is recorded on a verbal consent form and cosigned by the researcher obtaining consent and a witness.

#### Exclusion

Patients who are moribund, resident in a nursing home, with advanced dementia and high degree of dependency, or with end-stage frailty or terminal illness are excluded.

### Sample size

The sample size required to determine the independent associates of delirium and incident dementia is based on the rule of 10 outcomes per predictor. With the assumption of 10 independent predictors of delirium, 100 cases of delirium are required. Assuming five independent predictors of incident dementia, at least 50 dementia cases are required by end of 3-year follow-up. One previous study among general medical patients found a dementia occurrence of 60% in those with delirium over a median follow-up of 32.5 months (18.1% per year).^11^ Therefore, 83 people with delirium without dementia at baseline are required for our study. It is estimated that around half of patients with delirium have underlying dementia (with or without prior formal diagnosis),^12^ hence we would require 166 (83×2) patients with delirium at baseline. Based on our previous work showing a delirium occurrence of 30% in acute medicine patients aged >65 years, we will require an overall cohort size of 553 patients.^2^

### Study procedures

**Figure 2:**
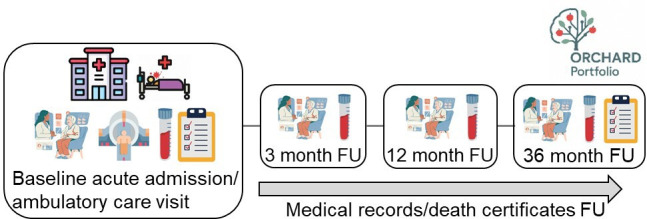
Schematic diagram of ORCHARD-PS participant recruitment, assessment and FU. FU, follow-up; ORCHARD-PS, Oxford and Reading Cognitive Health After Recovery from acute illness and Delirium—Prospective Study.

Participants are assessed at baseline recruitment (usually in hospital) and followed up in person at 3 months, 1 year and 3 years after recruitment by trained researchers (table 1, figure 2). A structured research proforma is completed using information gathered from participant interview supplemented with hand searching of entries in the medical records made by healthcare professionals during routine clinical care and from interviews with next-of-kin. In addition, information from each participant’s study proforma will be linked to their individual patient record in the Oxford and Reading Cognitive Comorbidity, Frailty and Ageing Research Database-Electronic Patient Records (ORCHARD-EPR).^13^ On enrolment, data collected include baseline demographics, admission diagnosis, medical history (including family history and sensory deficits), current medications, lifestyle, risk factors, residence and care needs.

**Table 1:** Summary of data collection at baseline and at follow-up (3 months, 1 year and 3 years)

Category	Variable	Visits			
		Baseline	3-month F/U	1-year F/U	3-year F/U
Recruitment	Informed consent or consultee declaration	✓	✓*	✓*	✓*
Demographic	Age, sex, ethnicity, postcode, socioeconomic class, education level, occupation, marital status and children, caring responsibility	✓	–	–	–
	Residence	✓	✓	✓	✓
Admission	Presenting complaint	✓	–	–	–
	Source of referral (ED, GP, SDEC)	✓	–	–	–
Medical history	CCI, Elixhauser Comorbidity Index, past medical history including dementia, depression, previous delirium	✓	–	–	–
Family history	Dementia, stroke, heart disease, hypertension	✓	–	–	–
Psychological background†	Subjective report of mood or changes in memory	✓	✓	✓	✓
	GDS	X	✓	✓	✓
Lifestyle†	Smoking history, alcohol intake, driving	✓	✓	✓	✓
Frailty	CFS, HFRS	✓	✓	✓	✓
	Long-term catheter, history of falls, hearing or vision impairment†	✓	✓	✓	✓
Functional status‡	Care needs, Barthel Index of ADL, mRS	✓	✓	✓	✓
	HABAM	✓	✓	✓	✓
	Nottingham Extended ADL Index, TUG	X	✓	✓	✓
Clinical assessments	HR, heart rhythm, BP including postural, Pain	✓	✓	✓	✓
Illness severity	NEWS, SIRS	✓	X	X	X
Nutrition	MUST, BMI	✓	X	X	X
Pressure sore risk	Braden Score	✓	X	X	X
Falls risk	Falls risk status	✓	X	X	X
Cognitive tests	AMTS, MoCA§, 4AT	✓	✓	✓	✓
	Reason cognitive test not done	✓	✓	✓	✓
	MMSE	X	✓	✓	✓
Delirium assessment and diagnosis	MDAS, CAM-S, OSLA, RASS	✓	–	–	–
	Delirium diagnosis (DSM-V)- prevalent vs incident, date of onset and subtype, and cause of delirium	✓	–	–	–
	Subsyndromal delirium diagnosis	✓	–	–	–
Diagnosis	Working diagnosis and final diagnosis	✓	–	–	–
	ICD-10 diagnosis codes	✓	–	–	–
Medication	Antipsychotics, benzodiazepines or other medication for behaviour management during admission	✓	–	–	–
	Current medication	✓	✓	✓	✓
In-hospital complications and outcomes	New catheter insertion and indication, new constipation, new pressure sore, inpatient fall, length of stay, delayed transfer of care	✓	–	–	–
Disposition	Discharge destination (usual residence, community hospital, hub bed, residential home or nursing home)	✓	–	–	–
	Care needs and care package	✓	✓	✓	✓
	Readmission to hospital within 30 days of discharge	–	✓	✓	✓
Neuropsychological battery	Boston Naming Test, Hopkins Verbal Learning Test, Rey Figure Copy, Symbol Digit Modalities Test, Trail Making Test A&B, Semantic Fluency	X	✓	✓	✓
Quality of life	EuroQol-EQ-5D-3L	X	✓	✓	✓
Informant assessment	IQCODE, NPI-Q	✓	X	X	✓
Laboratory tests	Blood banking	✓	✓	✓	✓
Brain imaging¶	CT- (or MRI-) brain scan (clinical or research)	✓	X	X	X
Mortality	During admission, on-follow-up	✓	✓	✓	✓
Long-term cognitive health outcomes	Cognitive test scores	✓	✓	✓	✓
	New dementia (and subtype) or mild cognitive impairment diagnosis	✓	✓	✓	✓
	New delirium	✓	✓	✓	✓
	Referral to memory clinic	✓	✓	✓	✓

* Review for regain of mental capacity to consent to research for participants recruited via consultee, or review of mental capacity for ongoing participation if any new concerns over lack of capacity.

† Review for new hearing or visual impairment and falls, change in smoking habit, alcohol consumption, ability to drive, mood and memory at follow-up visits.

‡ Baseline functional assessment is based on preadmission functional ability. Baseline HABAM is performed during admission.

§ Different versions of MoCA for each follow-up visit.

¶ Research CT brain scan is performed for participants who have not had a CT brain scan within 1 year prior to recruitment. Photon-counting CT brain scan is used for OUHFT participants, standard CT for RBFT participants.

ADL, activities of daily living; AMTS, Abbreviated Mental Test Score; 4AT, 4A’s test; BMI, body mass index; BP, blood pressure; CAM-S, Confusion Assessment Method- Severity Scale; CCI, Charlson Comorbidity Index; CFS, clinical frailty scale; DSM-5, Diagnostic and Statistical Manual of Mental Disorders, Fifth Edition; ED, emergency department; F/U, follow-up; GDS, geriatric depression scale; GP, general practitioners; HABAM, hierarchical assessment of balance and mobility; HFRS, Hospital Frailty Risk Score; HR, heart rate; IQCODE, Informant Questionnaire on Cognitive Decline in the Elderly; MDAS, Memorial Delirium Assessment Scale ; MMSE, Mini-Mental State Examination; MoCA, Montreal Cognitive Assessment; mRS, modified Rankin Scale; MUST, Malnutrition Universal Screening Tool; NEWS, National Early Warning Score; NPI-Q, Neuropsychiatry Inventory Questionnaire; OSLA, Observational Scale of Level of Arousal; RASS, Richmond Agitation Sedation Scale; SDEC, same day emergency care (ambulatory care service); SIRS, systemic inflammatory response syndrome; TUG, timed up & go test.

### Cognitive assessments at baseline

In the OUHFT and RBFT, cognitive screening including for delirium is mandated for all patients aged >65 years as part of standard care and is usually administered on admission by the clerking resident doctor (or occasionally the advanced nurse practitioner) via a structured proforma.^2 14^ The cognitive screen consists of the 10-point Abbreviated Mental Test Score (AMTS)^13^ combined with the short Confusion Assessment Method (CAM)^15^ or the 4A’s test (4-AT)^16^ together with documentation of delirium diagnosis and any pre-existing dementia.^2^ An AMTS <9 indicates cognitive impairment.^17 18^ The 4-AT is a delirium screening tool, with a score of 0 indicating that delirium or severe cognitive impairment is unlikely, score of 1–3 indicating possible cognitive impairment and score of 4 indicating possible delirium with/without cognitive impairment.

After enrolment into ORCHARD-PS, the research team undertakes further cognitive testing. The AMTS, 4AT and CAM are completed if not already done by the usual care team or if the patient’s clinical condition has changed. Delirium severity is characterised using the Memorial Delirium Assessment Scale (score of >13 indicates delirium, maximum score=30)^19^ and the long form of the CAM (maximum score=19),^20^ with a higher score indicating more severe delirium. The level of alertness, agitation and arousal is determined using the Richmond Agitation Sedation Scale and Observational Scale of Level of Arousal.^21^,^23^ These assessments are repeated every 48–72 hours by the research team where the patient remains in hospital to examine cognitive change over time including occurrence of incident delirium.

Participants also receive the Montreal Cognitive Assessment (MoCA) to measure the severity of cognitive impairment (maximum=30, ≥26=normal, 18–25=mild cognitive impairment, 10–17=moderate impairment and 0–9=severe impairment).^24^ The MoCA is performed as soon as possible after enrolment with the other assessments except where the patient is too unwell or has delirium in which case it is delayed until it becomes feasible. In addition to the usual item scoring, additional information is recorded including immediate recall, cued recall and the total number of words recorded in 1 min for the verbal fluency test and the total time taken to perform the test. Problems with testing (eg, visual or hearing impairment, poor English, aphasia) are documented.

### Delirium ascertainment

Delirium diagnosis is made by the principal investigator (STP) in discussion with the research team at weekly lab meetings and after review of all study records, clinical records and information from informants in accordance with the DSM-V criteria. Participants who have some but not all of the DSM-V criteria are classified as subsyndromal delirium.

Delirium is defined by onset time as described previously^2^:

- Prevalent: present on or within 48 hours of admission;
- Incident: developing at least 48 hours after admission;
- Any: presence of prevalent or incident delirium.
- Both: where patients with prevalent delirium have a further episode of incident delirium at least 48 hours after resolution of the previous delirium episode.

The duration of delirium is defined as the day of delirium diagnosis to the day of full resolution. Motor subtype is characterised as hypoactive, hyperactive or mixed. The aetiology of delirium is determined and may include one or more of infection, constipation, dehydration, medication, pain, new intracerebral event or other. The presence of pre-existing dementia diagnosis is used to classify delirium as delirium only versus delirium superimposed on dementia.

### Preadmission cognitive function and dementia

Participants or their consultee will nominate a suitable informant, who has known them for >10 years to complete two informant questionnaires. First, the 16-item Informant Questionnaire for Cognitive Decline in the Elderly (IQCODE), which assesses for preadmission cognitive impairment in which a score of >3.6 indicates dementia.^25^ Second, the 12-item Neuropsychiatric Inventory Questionnaire (NPI-Q)^26 27^ for the presence and severity of neuropsychiatric symptoms. Diagnosis of dementia will be made by STP according to the DSM-V criteria after review of all study and clinical records, IQCODE and informant report where relevant as described previously.^28^ Accepted criteria will be used to subtype dementia where possible as Alzheimer’s disease (eg, National Institute on Aging and the Alzheimer’s Association^29^), vascular dementia (eg, VasCog,^30^ VICCCS,^31^ NINDS-AIREN^32^), mixed, Parkinson’s disease dementia or Lewy body disease (Consortium criteria)^33^ or other.

### Physical function

Participants’ preadmission functional ability prior to acute illness is assessed using the modified Rankin Scale (mRS)^34 35^ and the Barthel Index for Activities of Daily Living (ADL).^36^ Frailty will be rated using the Clinical Frailty Scale as 2 weeks prior to admission or before the onset of the current acute illness.^37^ The Hierarchical Assessment of Balance and Mobility will be used to determine physical function in hospital and thereafter.^38^

### Comorbidity burden

We will collect information on comorbid disease from participant interview, hospital medical records including primary care records. Comorbidity burden will be calculated using the updated weighting of the Charlson Comorbidity Index^39 40^ and Elixhauser Comorbidity Index.^41^

### Clinical covariates

Vital observations, including blood pressure, heart rate, oxygen saturation, temperature and respiratory rate will be extracted from ORCHARD-EPR.^13^ Participants will be asked to rate their pain using a 10-point pain scale and the Wong-Baker FACES pain rating scale.^42^ The Malnutrition Universal Screening Tool^43^ is used to determine the risk of malnutrition, pressure sore risk is measured using the Braden Scale^44^ and falls risk is assessed using standard hospital protocol by the clinical care team.

### Blood biomarkers

We will extract baseline routine laboratory results (eg, white cell count, neutrophil/lymphocyte ratio, C reactive protein (CRP) level, microbiology) from ORCHARD-EPR.

Additional research blood samples (approximately 20 mLs) are collected for blood banking and storage at −80°C as follows:

- Cytodelics-stabilised whole blood for flow cytometry.
- Whole blood (one 1.5 mL and three 500 µL samples).
- Buffy coat.
- EDTA plasma (three 500 µL samples).
- Lithium-heparin plasma (three 500 µL samples).
- Serum (three 500 µL samples).
- Tempus blood RNA tube.

Future biomarker analyses will include:

- Flow cytometry for differential immune cell counts and feature characteristics.
- Cytokine panel (n ~40, eg, Luminex assay).
- CNS markers (plasma amyloid, τ, neurofilament light chain, glial fibrillary acidic protein, serum 100 beta protein, neurogranin).
- Proteomics (eg, Olink, ~5000 proteins).
- Transcriptomics.
- DNA extraction and Apolipoprotein E genotyping.

Blood sampling is performed at baseline and at each follow-up visit.

### Neuroimaging biomarkers

Brain imaging (CT or MRI scan) completed as part of routine clinical care during the index admission or previous clinical encounters is obtained from the hospitals’ Picture Archiving and Communication System and pseudonymised (deidentified) for analysis. For participants who do not have existing brain imaging, a research CT-brain scan is performed. Participants from OUHFT requiring a research scan undergo brain imaging using the Acute Multidisciplinary Imaging and Interventional Centre photon-counting CT-(PCCT) scanner situated in the John Radcliffe Hospital, Oxford, whereas RBFT participants have a standard CT-brain scan. Incidental findings are communicated to participants as soon as possible and to their general practitioners and relevant clinical teams with the participants’ permission.

We will quantify the severity of brain atrophy and white matter disease using validated visual rating scales including the Global Cortical Atrophy Scale^45^ and the Fazekas^46^ and/or Age-Related White Matter Changes Scale.^47^ The presence of other brain lesions (eg, stroke or tumour) will be recorded.

### Follow-up assessments (3-month, 1-year and 3-year)

Participants are followed up in-person at the research clinic or by home visit or telephone if necessary. A structured proforma similar to the baseline assessment proforma is used, including information on health condition, frailty, residence and care needs. We also collect information on any intervening hospital admissions or attendance at memory or falls clinics. A brief clinical examination measuring pulse, blood pressure (and postural blood pressure) is performed. The IQCODE and NPI-Q will be repeated at 3-year follow-up.

Indirect follow-up using ORCHARD-EPR^13^ (which includes linked mental health hospital records) and hand searching of medical records including primary care records via the hospital EPR portals will be used to mitigate loss-to-study-interview follow-up. Data on all-cause mortality will be obtained from the Office of National Statistics.

#### Cognitive assessments at follow-up

Cognitive assessments including AMTS, 4AT, MoCA and Mini Mental State Examination^48^ will be undertaken. Additional cognitive testing including a short Neuropsychological Battery Test is done tailored to participants’ ability including the Boston Naming Test,^49^ Hopkins Verbal Learning Test,^50^ Rey Figure Copy,^51^ Symbol Digit Modalities Test,^52^ Trail Making Test A and B^53^ and Semantic Fluency.^54^

#### Physical function

ADL are assessed using the mRS and Barthel Index. Additionally, participants complete the Nottingham ADL scale^55^ and the Timed Up and Go test.^56^

#### Quality of life and mood

Participants complete the EuroQol-EQ-5D-3L^57^ to measure quality of life and the Geriatric Depression Scale to screen for depression.^58^

#### Participant evaluation questionnaire

A mixed quantitative and qualitative questionnaire is administered at 3-month follow-up and will be repeated at the 3-year follow-up (see online supplemental file 1). Participants are asked about their experience of cognitive assessments in hospital, whether they would wish to be informed of their dementia risk in future according to any risk prediction algorithms in development, and their preferred method of being advised on brain health and dementia risk (eg, informed by hospital staff vs general practitioners vs via written information only).

### Outcome measures

The outcome measures include:

- Length of stay defined as day of admission to day of discharge from OUHFT and RBFT acute care.
- Change in care needs defined as change in frequency of care package or number of carers (single-handed vs double-handed), discharge to community hospital or rehabilitation unit or new discharge to care home or nursing home.
- Institutionalisation defined as new permanent placement at care home or nursing home.
- Mortality during admission or during follow-up period. We will also record cause of inpatient death.
- Readmission to hospital within 30 days of discharge.
- New dementia diagnosis and subtype (Alzheimer’s disease, vascular dementia, mixed, Parkinson’s disease dementia, Lewy body dementia or other).
- Longitudinal change in cognition and functional status.

Table 1 summarises the data collected prospectively at baseline and at follow-up (3 months, 1 year and 3 years).

### Linkage to ORCHARD-EPR

Baseline and follow-up participant interview data will be supplemented through linkage to ORCHARD-EPR.^13^ ORCHARD-EPR contains structured information from assessments performed by the healthcare team as part of standard care including nursing risk assessments (falls risk, nutrition, pressure sore risk, frailty), cognitive assessments (10-point-AMTS, CAM,^15^ 4A’s test-4AT,^16^ MoCA), observations, laboratory test results and hospital administrative information including ICD-10 diagnostic and procedure coding and outcomes including length of stay, discharge destination and mortality. Derived variables include illness severity (systemic inflammatory response syndrome-SIRS, National Early Warning Score-NEWS2)^59 60^ and the Hospital Frailty Risk Score.^61 62^

### Statistical analyses

Demographic data will be summarised using descriptive statistics and comparison between delirium versus no delirium groups will be performed using the χ^2^ test for categorical data and t-test/analysis of variance for continuous data. The prevalence and incidence of delirium, including the subtype (hypoactive, hyperactive, mixed), and the severity and duration of delirium will be determined together with the prevalence of preadmission dementia diagnosis and incidence of new dementia diagnosis on follow-up including the subtype. Logistic regression will be used to determine the associates of delirium (any, and by subtype and severity) adjusted for covariates including demographic factors, infection, illness severity, baseline cognition, frailty and comorbidity and apolipoprotein E genotype.

Cox proportional hazards regression will be used to determine the HRs for new dementia on follow-up in participants with versus without delirium, adjusted for covariates as above and stratified by baseline brain imaging factors (small vessel disease, atrophy). Longitudinal cognitive data from all time points will be used to examine the evolution of cognitive impairment over time including in specific cognitive domains. Free text responses in the evaluation questionnaire will undergo thematic analyses to determine participants’ experience and understanding of the rationale for performing cognitive tests in hospital, and their perspectives on being informed about their brain health and the risk of developing dementia in future based on information collected in their hospital records.

### Patient and public involvement

Our patient and public involvement (PPI) group includes patients with lived experience of cognitive frailty and acute hospital admission, carers, a retired general practitioner and members of the Alzheimer’s Society. The PPI group previously highlighted the need for studies to inform the development of delirium treatments and for better information for patients and carers on the prognosis of delirium, particularly in identifying those at higher risk of developing dementia. Group members provided input into the ORCHARD-PS protocol, the evaluation questionnaire and study design including around potential logistical challenges for patients returning for follow-up assessments at the hospital. The protocol therefore includes the option of telephone and home visit follow-up where required. Going forward, the PPI group will inform the conduct, outcome selection, reporting and dissemination of the study.

## Discussion

### Summary

ORCHARD-PS is a bi-centre, observational study of the impact of acute illness and delirium on cognitive health and future dementia risk including detailed characterisation of delirium and collection of clinical, blood and neuroimaging biomarkers. Data are collected from a range of sources, including direct assessment and interview with participant or informant supplemented by electronic medical record linkage and hand searching of records. ORCHARD-PS focuses on older hospital patients who are most vulnerable to delirium and includes people across the frailty spectrum who are generally under-represented in research.^63^ Through the use of multimodal biomarkers, ORCHARD-PS will advance our understanding of delirium pathophysiology necessary to develop new treatments and the underpinning mechanisms linking delirium and acute illness to future dementia.

### Biomarkers of the systemic response

Acute illness induces inflammatory, immunologic and other systemic responses, which are thought to precipitate delirium in many cases. It is hypothesised that peripheral inflammatory cytokines cross the blood–brain barrier and trigger an inflammatory cascade in the brain, resulting in neuronal injury and cognitive dysfunction.^64 65^ Inflammatory mediators are the most frequently studied biomarkers in delirium, particularly IL-6, CRP, tumour necrosis factor-α and IL-1β, although results are conflicting possibly owing to differences in patient populations, small sample sizes and adjustment for confounders.^10 66^ Most studies are focused on surgical or critically ill patients, with limited data from older acute medical inpatients^67^ where inflammatory biomarker profiles may vary with different predisposing and precipitating factors.

### CNS biomarkers

The cognitive impact of delirium extends beyond the immediate period of acute illness and is associated with long-term cognitive decline. Several mechanisms are thought to contribute to neuronal injury or damage, including neuroinflammation, altered brain energy metabolism including impaired glucose utilisation^68^ or activation of the kynurenine pathway with increased levels of neurotoxin quinolic acid.^69^ Examining biomarkers of neuronal function in alignment with The National Institute on Aging-Alzheimer’s Association biological construct of ATN biomarkers for Alzheimer Disease and related dementias may help inform underlying brain vulnerability including the presence of pre-existing neurodegeneration and subsequent dementia risk. Delirium has been associated with such biomarkers,^70 71^ but there remains uncertainty whether this simply indicates the presence of pre-clinical dementia or whether delirium initiates an acute neurodegeneration process. Recently, neurofilament light chain (a marker of neuronal injury) was shown to increase in acute illness and be persistently elevated at follow-up, suggesting precipitation of neuronal damage.^71^ Interestingly, neurofilament changes were greatest in those with a better cognitive baseline consistent with previous studies demonstrating greater cognitive impacts of delirium in those with good cognition.^5 71 72^ In other studies, S100Beta (a marker of blood–brain barrier permeability) has shown conflicting associations with delirium.^73^

It has been proposed that there are distinct pathophysiological pathways underlying delirium aetiologies and subtypes linked to diverse predisposing and precipitating factors (eg, infection, dehydration, liver failure, hypoxia, medication). However, there are few existing studies comparing biomarker profiles between delirium subtypes, and in most, sample sizes are too small for meaningful analysis.

### Neuroimaging

Structural brain changes such as white matter disease and atrophy are associated with increased delirium risk^74^ and cerebrovascular disease^75^ may be particularly important. MRI is the most commonly used imaging modality in neuroscience research, owing to lack of radiation exposure and high levels of tissue contrast and spatial resolution. However, MRI is expensive and challenging to perform in older confused patients in the acute hospital setting and only feasible in a subset of patients. In contrast, CT brain scanning is available as part of standard care for around 60% of older hospital patients either from the index admission or from previous encounters and around ~15% of these also have MRI.^76^

In ORCHARD-PS, we have taken a pragmatic decision to exploit brain imaging acquired as part of standard care, either CT or MRI, and to supplement this with research brain scans, currently CT-based, to obtain brain imaging for as many participants as possible and avoid bias by indication. A recently developed technology, PCCT, offers greater spatial resolution and the potential for better grey–white matter differentiation compared with conventional CT^77^ and is currently used for ORCHARD-PS research brain imaging for Oxford participants. The use of PCCT in our study will help better understand its added value over conventional CT in the older population at risk of delirium and dementia in whom MRI may not be feasible.

### Knowledge gaps and insights from related areas

Current delirium biomarker studies are limited by a focus on one, or at most a few, specific biomarkers of the systemic response, neuronal injury or brain imaging. Studies combining multimodal biomarkers informative for both the systemic response and the underlying brain status in delirium and future dementia are therefore required to provide insights into molecular pathways and systemic/brain interactions. The importance of brain imaging is illustrated by our recent observation that white matter disease modifies associations between infection and future dementia risk but not delirium-dementia risk associations, indicating that different mechanisms may link particular acute illness features to specific dementia subtypes. Multiomics and machine learning approaches to large-scale multimodal data^78^ may help identify feature groups at increased risk of delirium and dementia but have not yet been widely explored.^79 80^ Proteomics^81^ and genome-wide association studies (GWAS) implicate innate immune, metabolic, synaptic and vascular homeostasis pathways in dementia and also in proteomic ageing clocks.^82^,^84^ Overlap between omics findings in dementia and hallmarks of ageing^85^ and delirium might therefore be expected and is supported by preliminary GWAS and proteomics studies.^79 86^

### Strengths and limitations

Strengths of our study include the longitudinal design with prospective delirium ascertainment including in patients lacking capacity to consent to research and careful phenotyping enabling adequate adjustment for confounders, acquisition of multimodal biomarkers and multiple follow-up strategies to minimise selection and attrition bias. There are some limitations. First, daily delirium screening for all included patients is not possible owing to practical issues and resource constraints. However, regular real-time EPR review is undertaken by the study team including of daily ward round entries and nursing staff records for any acute changes in cognition minimising the chances of missing delirium. Second, although our study design mitigates loss to follow-up, it is nevertheless likely that some selective attrition of frail and multimorbid participants may occur. However, the use of indirect follow-up using medical records and death certificates will ensure some level of follow-up on all participants. Third, although blood samples are collected at baseline and at each follow-up visit, we do not perform serial blood collection during admission, and therefore we are unable to assess the temporal evolution of blood biomarkers between delirium onset and resolution. Fourth, our study is limited to the older population and our findings may not be generalisable to younger people or those from different settings.

In summary, ORCHARD-PS will provide a rich research resource for studies on the impact of acute illness on the ageing brain and specifically the mechanisms underlying delirium and future dementia risk. Findings will address important knowledge gaps and help identify potential therapeutic targets for delirium and dementia treatment and prevention.

## Ethics and dissemination

ORCHARD-PS is approved by the South Central- Berkshire Research Ethics Committee (REC Reference: 23/SC/0199). Results will be disseminated through peer-reviewed publications and conference presentations and lay interest groups. Anonymised data will be made available to external researchers at project end, which will be defined as the completion of publications by the study team.

## Supplementary material

10.1136/bmjopen-2025-102028 online supplemental file 1
