# Supplementary material for: Protocol for a prospective cohort study to determine the multimodal biomarkers of delirium and new dementia after acute illness in older adults: ORCHARD-PS
Source: BMJ Open. 2025 Jun 13;15(6):e102028. doi: 10.1136/bmjopen-2025-102028 (PMC12164623; doi:10.1136/bmjopen-2025-102028)
Supplement: online supplemental file 1 [file bmjopen-15-6-s001.docx]

**Protocol for a prospective cohort study to determine the multimodal biomarkers of delirium and new dementia after acute illness in older adults: ORCHARD-PS**

Jasmine Ming Gan^1^, Lily Elderton^1^, Meenu Vijayakumar Sheela^2^, Jessica Knight^3^, John Louca^3^, Sarah Evans^4^, Kinza Shahab^4^, Nicola G Lovett^4^, Mary Sneade^1^, Nycola Muchenje^5^, Mariya Fenchyn^5^, Davide Simonato^6^, Aubretia McColl^1,5^, Sarah T Pendlebury^1,4^

**^1^ Wolfson Centre for Prevention of Stroke and Dementia, Wolfson Building, Nuffield Department of Clinical Neurosciences, John Radcliffe Hospital, University of Oxford, Oxford, UK**

**^2^ Neurosciences Research Delivery Team (DENDRON), Oxford University Hospitals NHS Foundation Trust, Oxford, UK**

**^3^ Oxford (Thames Valley) Foundation School, Oxford University Hospitals NHS Foundation Trust, Oxford, UK**

**^4^ Departments of Acute General Internal Medicine and Geratology, Oxford University Hospitals NHS Foundation Trust, Oxford, UK**

**^5^ University Department of Elderly Care, Royal Berkshire NHS Foundation Trust, Reading, UK**

**^6^ Department of Neuroradiology, Oxford University Hospitals NHS Foundation Trust, Oxford, UK**

**Supplementary Methods**

**Development of the participant evaluation questionnaire**

The participant evaluation questionnaire was developed in partnership with the ORCHARD Patient and Public Involvement (PPI) group. The questionnaire includes both multichoice questions and free text responses including from family/carers where appropriate but in-depth interviews are not performed. The PPI group reviewed the first draft of the questionnaire and made changes to the language to ensure it was lay friendly and suggested additional questions to be included. The resulting questionnaire was tested on the first 50 participants and the responses were shared with the PPI group, resulting in further revisions to the phrasing of questions.

ORCHARD-PS Participant ID:
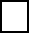

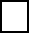
 -
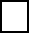

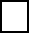

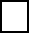

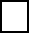
 Date: ____ ____ ____

Participant evaluation at 3 months/3 years* * delete as appropriate

(Questions to be asked by the researcher)

1. ***When you were last admitted to hospital, you underwent some brief assessments to test your thinking and memory. We do this for all older people whatever the reason for their admission to hospital.***

What was your experience of having assessments of thinking and memory in hospital?

Tick all that apply:

Did not remember the tests being done

Thought it was important

Didn’t understand why it was necessary

Found the tests tiring

Found the tests interesting/enjoyed the challenge

*Please add any other comments below:*

**2.** Do you know why hospital staff do brief tests of thinking and memory?

*Please write the participant’s answer below:*

***When you were in hospital, staff entered a lot of information about you and your health into the computer records. These computer records could be used to tell you about your brain health over the next few months and years.***

**3**. After being admitted to hospital, would you want to know about your current brain health?

YES/NO/Uncertain

**4.** Would you want to know whether you have a low or high chance of developing dementia?

YES/NO/Uncertain

**If YES, to questions 3 & 4 please answer questions 5, 6 7 and 10 only**

**If NO or Uncertain to questions 3 & 4, please answer questions 8, 9 and 10 only**

**IF YES:**

**5.** Why would you want to be informed about your brain health and the chance of dementia/deterioration in the future?

*Please write the participant’s comments below:*

**6.** It is unlikely that we could ever be completely certain about a person’s brain health and the chance of developing dementia/deterioration in the future. Given this uncertainty, would you still prefer to be told you had a chance of developing dementia (low or high risk)?

YES/NO/Uncertain

**7.** Who would you prefer to inform you about your brain health and dementia/deterioration risk

(tick one):

i) A discussion with hospital staff looking after you during your time in hospital?

ii) A copy of the hospital discharge letter sent to your GP?

iii) Having a discussion with your GP after leaving hospital?

iv) Other ……………………………………………..

**If NO/Uncertain:**

**8.** Would your view change if you knew hospital staff or your GP would provide you with information about how you could help keep your brain healthy in the future?

YES/NO/Uncertain

**9**. Would your view change if we had a treatment that might reduce your chance of developing dementia/deterioration in the future?

YES/NO/Uncertain

**10. Any other comments** (including your family or carer’s views) about thinking and memory tests in hospital? Should we use information from hospital records to tell people about brain health and the chance of developing dementia/deterioration in the future?

*Please write any other comments below:*

Participant

Family member/carer

**Researchers only:** *If form not done or incomplete please give reason:*
