# Supplementary material for: Protocol for a prospective cohort study to determine the multimodal biomarkers of delirium and new dementia after acute illness in older adults: ORCHARD-PS
Source: BMJ Open. 2025 Jun 13;15(6):e102028. doi: 10.1136/bmjopen-2025-102028 (PMC12164623; doi:10.1136/bmjopen-2025-102028)
Supplement: Supplementary file 2 [file bmjopen-2025-102028.reviewer_comments.pdf]

PEER REVIEW HISTORY

BMJ Open publishes all reviews undertaken for accepted manuscripts. Reviewers are asked to complete a checklist review form and are provided with free text boxes to elaborate on their assessment. These free text comments are reproduced below.

ARTICLE DETAILS

Title (Provisional)

Protocol for a prospective cohort study to determine the multimodal biomarkers of delirium and new dementia after acute illness in older adults: ORCHARD-PS

Authors

Gan, Jasmine Ming; Elderton, Lily; Sheela, Meenu Vijayakumar; Knight, Jessica; Louca, John; Evans, Sarah; Shahab, Kinza; Lovett, Nicola G; Sneade, Mary; Muchenje, Nycola; Fenchyn, Mariya; Simonato, Davide; McColl, Aubretia; Pendlebury, Sarah

---

This article was not externally reviewed at BMJ Open. Protocols that have been independently assessed prior to submission to BMJ Open are usually fast-tracked to publication on the grounds that further substantial changes will not be possible. This independent assessment will usually be external, independent review for both a substantial grant award from a non-commercial or government funder and ethics approval.
